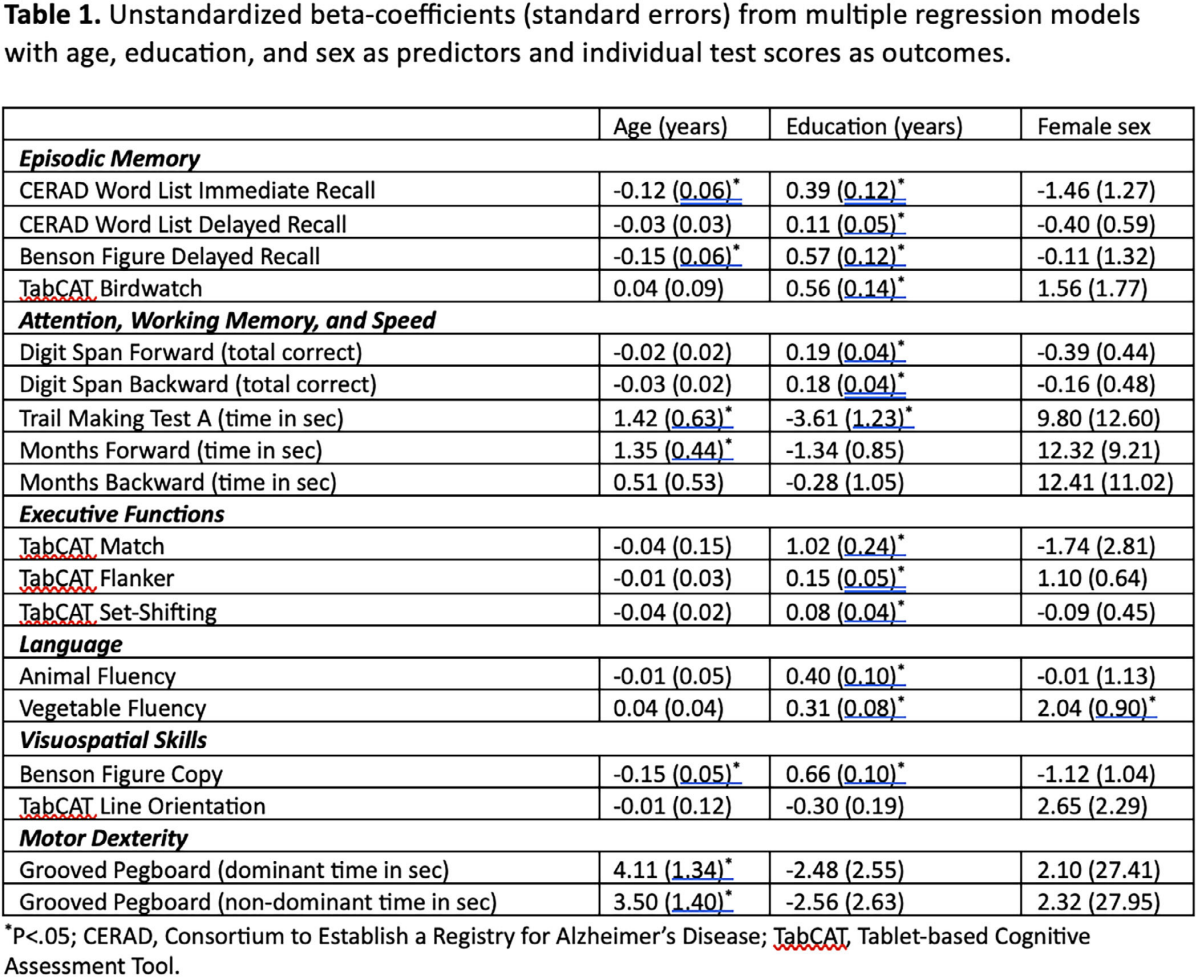# Designing Culturally Appropriate Cognitive Tools in Resource Limited Setting: Lessons from Ethiopia's Multilingual and Culturally Diverse Context

**DOI:** 10.1002/alz70857_102389

**Published:** 2025-12-25

**Authors:** Yared Z Zewde

**Affiliations:** ^1^ College of Health Sciences, Addis Ababa University, Addis Ababa, Ethiopia; Global Brain Health Institute, San Francisco, CA, USA

## Abstract

**Background:**

Rising prevalence of Alzheimer's disease and related dementias (ADRD) in Sub‐Saharan Africa (SSA) highlights an urgent need for effective cognitive assessment tools tailored to this region. Existing tools are often culturally and linguistically inappropriate and have inadequate validity and reliability. We report findings from Ethiopia on adapting cognitive tools incorporating local cultural and social contexts. Our work aims to identify challenges in adapting widely used ADRD cognitive assessments in Ethiopia, and explore social, cultural, and linguistic factors influencing cognitive performance.

**Method:**

Using a mixed‐methods approach, we evaluated the cultural applicability of widely used paper‐and‐pencil and digital cognitive tools in urban Ethiopian setting. The study incorporated ethnographic observations, focus group discussions with local communities and normative data development. By July 2024, 100 healthy older adults (age: 62.1±9.1, 60% female, education: 8.0±5.5) who met the inclusion and exclusion criteria harmonized with the National Alzheimer's Coordinating Center protocols were enrolled. Qualitative thematic analysis and multiple linear regression models were used to present the results.

**Result:**

Thematic analyses indicated challenges related to high rates of screening failures (visual deficits and subjective memory complaints), negative perception of cognitive problems, and limited literacy and technology familiarity. Floor effects were identified on tasks of processing speed (Trails A = 16%, Months Backward = 11%) and motor dexterity (Grooved Pegboard = 10%). Multiple regression analyses indicated strong effects of educational attainment followed by age across most tasks, while female sex effect was present only on task of language functions (Table 1).

**Conclusion:**

The study emphasizes the important need for culturally adapted cognitive tools in Ethiopia. Although low literacy, stigma, and limited technology understanding pose challenges, our finding support accessibility and feasibility of neuropsychological tools for ADRD research by incorporating local languages, culturally relevant tasks, and literacy‐independent assessments. Our findings inform development of culturally valid cognitive protocols by highlighting best performing internationally harmonized tasks and identifying predictors of performance. Based on our learnings, we will provide recommendations on the design of aging and ADRD studies in low resource settings in SSA including strengths and weakness of harmonized cognitive assessment protocols.